# Recent and Frequent Vertigo Attacks Produce Negative Findings on Furosemide-Loading Vestibular Evoked Myogenic Potential Testing in Meniere's Disease

**DOI:** 10.3389/fneur.2018.00636

**Published:** 2018-08-03

**Authors:** Toru Seo, Ko Shiraishi, Takaaki Kobayashi, Takeshi Fujita, Kazuya Saito, Katsumi Doi

**Affiliations:** Department of Otolaryngology, Kindai University, Osakasayama, Japan

**Keywords:** furosemide, vestibular evoked myogenic potential (VEMP), endolymphatic hydrops, Meniere's disease, saccule, rupture of membranous labyrinth

## Abstract

**Objective:** The peak-to-peak amplitude of the p13-n23 wave in cervical vestibular evoked myogenic potential can increase after furosemide administration in patients with Meniere's disease [furosemide-loading VEMP (FVEMP) testing]. The examination is used to test for the presence of endolymphatic hydrops; we investigated factors that may influence the results.

**Methods:** Forty-two subjects (23 males and 19 females, aged 24–70 years) with unilateral definite Meniere's disease who underwent FVEMP testing were retrospectively studied. Possible factors associated with the results of FVEMP testing were studied using logistic regression analysis.

**Results:** Ages, sex, affected side, stage, disease duration, and mean hearing level of pure tone audiometry did not influence the results of FVEMP testing in the univariate analysis (*p* > 0.05). Number of days since the last vertigo attack [odds ratio (OR): 1.07, *p* = 0.031] and frequency of vertigo attacks per month (OR: 0.42, *p* = 0.003) were significantly associated with the results of testing. Multivariate analysis showed that both days since the last vertigo attack < 7 (OR: 0.13, *p* = 0.04) and frequency of vertigo attacks per month ≥ 2 (OR: 0.06, *p* = 0.004) were risk factors for negative results on FVEMP testing.

**Conclusion:** This study found that recent and frequent vertigo attacks produced negative findings on FVEMP testing in Meniere's disease. This apparently irrational finding can be explained by the consequences of membranous labyrinth rupture during vertigo attacks, where the altered saccular resonance due to EH cannot be recovered by furosemide administration because of the dissolving dehydration effect that occurs through communication between the endolymphatic and perilymphatic spaces. In addition, the impairment of sensory cells that is caused by endolymph and perilymph mixing upon rupture does not improve upon furosemide administration. FVEMP testing results may provide us with pathophysiological information regarding the membranous labyrinth.

## Introduction

The etiology of Meniere's disease (MD) is known to involve idiopathic endolymphatic hydrops (EHs) based on Yamakawa's human temporal bone study of 1938 (1). This pathological finding was only detected in the temporal bone specimen, which had been harvested after death. Approximately 70 years later, Nakashima et al. detected EHs *in vivo* using gadolinium (Gd)-enhanced magnetic resonance imaging (MRI) (2). This method should develop as a useful tool for the diagnosis of EH; however, it still can be used in limited institutions because it requires a 3T device. Thus, the following electrophysiological test and diuretic loading tests are considered acceptable for the detection of EHs.

Electrocochleography is an electrophysiological test that is used to detect EH (3). An action potential (AP) is a summed response of numerous, at times thousands of, auditory nerve fibers that fire synchronously. The summating potential (SP) is a complex response that is composed of several components. It is generated by the hair cells of the organ of Corti and a reflection of the displacement-time pattern of the cochlear potential. An increased endolymph volume creates mechanical biasing vibration of the organ of Corti; subsequently, the magnitude of SP enlarges. Thus, the SP/AP ratio provides a useful measure for detecting EH.

Next, some diuretic loading tests are based on the improvement of inner ear function of hydropic ear due to the dehydration of diuretic. First, the hearing threshold is improved with glycerol administration in patients with MD (4). This observation led to the development of the glycerol test, which may detect the presence of EHs in the cochlea. Second, the maximum slow-phase velocity of nystagmus during caloric testing and the gain of the vestibular-ocular reflex (VOR) during rotatory examinations increase after furosemide administration in patients with MD (5, 6). These observations are used in Futaki's furosemide test and the furosemide VOR test, which can indicate the presence of EHs in the semicircular canal. Moreover, the p13-n23 peak-to-peak amplitude of cervical vestibular evoked myogenic potentials (cVEMP) rises 60 min after furosemide administration in patients with MD [furosemide-loading VEMP (FVEMP) testing] (7, 8). As the cVEMP reflects saccular function, the examination is conducted to detect EHs in the saccule. The positive rate of FVEMP stimulated by click sound is 40% on MD. On the same series, the positive rate of glycerol test, Futaki's method and electrocochleography were 44, 40, and 46%, respectively. The effectiveness of various tests on Meniere's disease diagnosis is not very different. The FVEMP results were independent of the results of other tests (8). Because the peripheral organs that were evaluated during each examination were different, this result was acceptable.

There are alternate methods that are used to detect saccular EH and use glycerol as an osmotic diuretic. Murofushi compared the amplitudes of cVEMP 3 h after oral administration of glycerol (1.3 g/kg body weight) with those recorded before administration and found that the positive rate in patients with MD was 38% (9). Shojaku compared the amplitude of cVEMP 1 and 2 h after intra-venous administration of 500 mL of 10% glycerol with those before administration (10). In this case, the positive rate was 53% in patients with MD. Diuretic loading cVEMP using glycerol is equivalent to that using furosemide in MD diagnoses. FVEMP requires shorter examination time compared to the glycerol test, and glycerol VEMP and can be performed following Futaki's test. Owing to these favorable features, we mainly used FVEMP.

To revise the FVEMP, we studied cVEMP amplitudes that were stimulated at 250, 500, 700, 1,000, 1,500, and 2,000 Hz and were measured before and after furosemide administration in MD and normal control groups (11). The amplitudes after administration significantly increased at only 500 Hz in the subjects with MD. Thus, the revised FVEMP test was measured using a 500-Hz tone-burst sound stimuli. Receiver operating characteristic curve analysis revealed an IR cut-off value of 14.2% at 500 Hz, with a sensitivity of 71% and a specificity of 81% for comparing MD to normal healthy subjects. Owing to this revision, the examination was more acceptable for the diagnosis of EHs in the saccule (11). On the other hand, EHs were detected in the saccule in 86% of patients with MD based on a human histopathological study and in 93% of patients with MD based on an MRI study (12, 13). The FVEMP study detected a somewhat lower percentage of EHs than the pathological study and MRI study. The aim of this study was to examine the factors that influence the results of FVEMP testing in patients with MD.

## Materials and methods

Patients who underwent FVEMP testing at the Department of Otolaryngology, Kindai University Hospital, between October 2014 and March 2017 were enrolled. Among them, we selected subjects that were diagnosed with MD based on the criteria set by the Barany Society after the reconfirms of the patient's medical chart, and used the subjects in this study (14). They include previous prescription of anti-vertigo drugs or diuretics. The exclusion criteria were contralateral lesions, conductive hearing loss, vertigo-related lesions, including internal auditory meatal lesions; lack of audiological examination results, history of ear surgery, history of intratympanic drug administration, and refusal to participate in this study. The subjects' ages, sex, affected side, stage, disease duration, period since the last vertigo attack, frequency of vertigo attacks per month, and mean pure tone audiometry (PTA) in lower, middle, and higher frequencies were obtained from clinical charts. The clinical stage of the disease and the frequency of vertigo attacks per month were defined based on the guidelines developed by the American Academy of Otolaryngology-Head and Neck Surgery in 1995 (15). The staging is based on the four-tone average of the PTA at 500, 1,000, 2,000, and 3,000 Hz of the worst audiogram during the interval 6 months before treatment. Stage 1–4 means the four-tone average of ≤25 dB, 26–40 dB, 41–70 dB, and > 70 dB, respectively. PTA in lower, middle, and higher frequencies indicated an averaged hearing level at 125 and 250 Hz, 500, 1,000, and 2,000 Hz, and 4,000 and 8,000 Hz, respectively. The subjects consisted of 23 males and 19 females. Their ages ranged from 24 to 70 (mean: 51.0, SD: 12.4) years.

### Measuring FVEMP

The FVEMP examination procedure was conducted based on a previously reported method using the Neuropack system (Nihon-Kohden Co, Tokyo, Japan) (11). The active electrode was placed on the upper half of the sternocleidomastoid muscle, the reference electrode was placed on the upper manubrium sterni, and the ground electrode was placed on the forehead. Tone burst stimuli with 500 Hz (rise/fall time: 1 ms; plateau time: 4 ms) and 135 dB SPL were delivered to the ipsilateral ear via headphones at a 5 Hz repetition rate. During the recording procedure, the subjects lay in the supine position and were instructed to turn their heads away from the side on which the stimuli were delivered to ensure that the tonus of the sternocleidomastoid muscle remained constant. The original peak-to-peak amplitudes of cVEMP were recorded and then bandpass filtered (5 Hz to 1 kHz). The mean myogenic activity of the fully rectified wave detected during the 20 ms before the delivery of the stimuli was also recorded. To ensure that differences in muscular tonus did not affect the recording, normalized amplitudes were calculated by dividing the original amplitude by the mean myogenic activity. The normalized amplitude of VEMP was measured before and 60 min after the intravenous administration of 20 mg furosemide (16). As described in previous reports, the improvement rate (IR) was obtained using the following formula:

$$IR=100\;\times \;\frac{AA-AB}{AB}\;(\%)$$

Where AB was the normalized amplitude observed before the administration of furosemide and AA was the normalized amplitude seen after the administration of furosemide (6, 8). According to the previous study, the criteria for positivity were defined as IR > 14.2% or when the p13-n23 biphasic wave was detected only after the administration of furosemide (11). All subjects were prohibited from using diuretics and anti-vertigo drugs, which could affect the results, 24 h before examinations.

### Statistical analysis

Possible factors that could influence the FVEMP testing results were examined using stepwise logistic regression analysis. Factors with at least borderline significance (*p* < 0.20) according to the univariate analysis were included in the multivariate analysis. Differences in percentages between the two groups were analyzed using Fisher's exact test. Continuous variables between the two groups were analyzed with Student's *t*-test. All data were analyzed using EZR version 1.35 (Saitama Medical Center, Jichi Medical University, Saitama, Japan), which is based on the open-source statistical software R (R Foundation for Statistical Computing, Vienna, Austria) (17). *p*-values of < 0.05 were considered to be significant.

## Results

**Figure 1:**
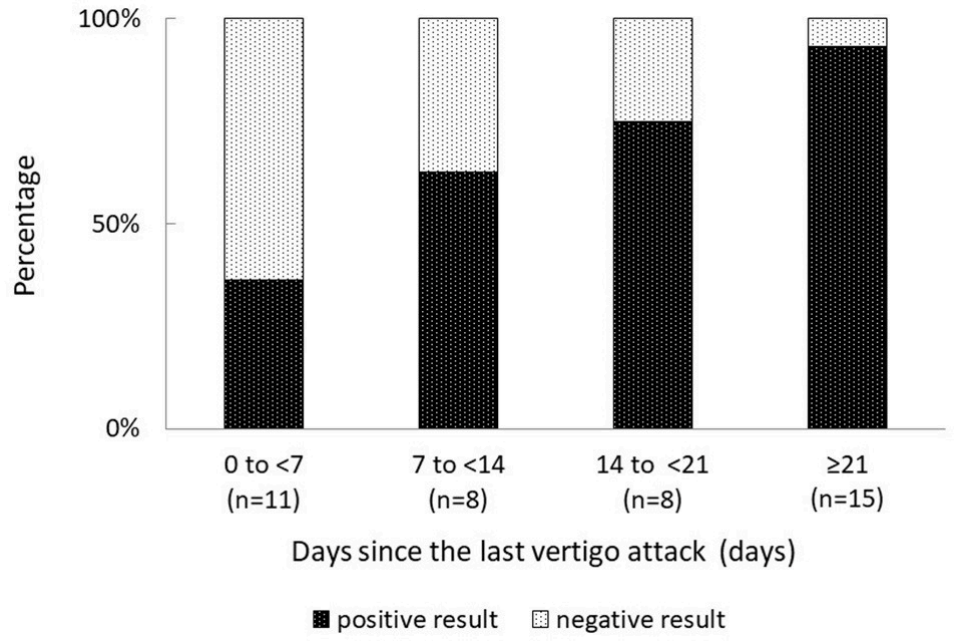
Negative results are observed in only 7% of patients in whom the last vertigo attack occurred ≥21 days previously, and in 64% of patients in whom the last vertigo attack occurred ≤ 7 days previously. The shorter the period since the last attack, the more likely that the examination results would be negative.

**Figure 2:**
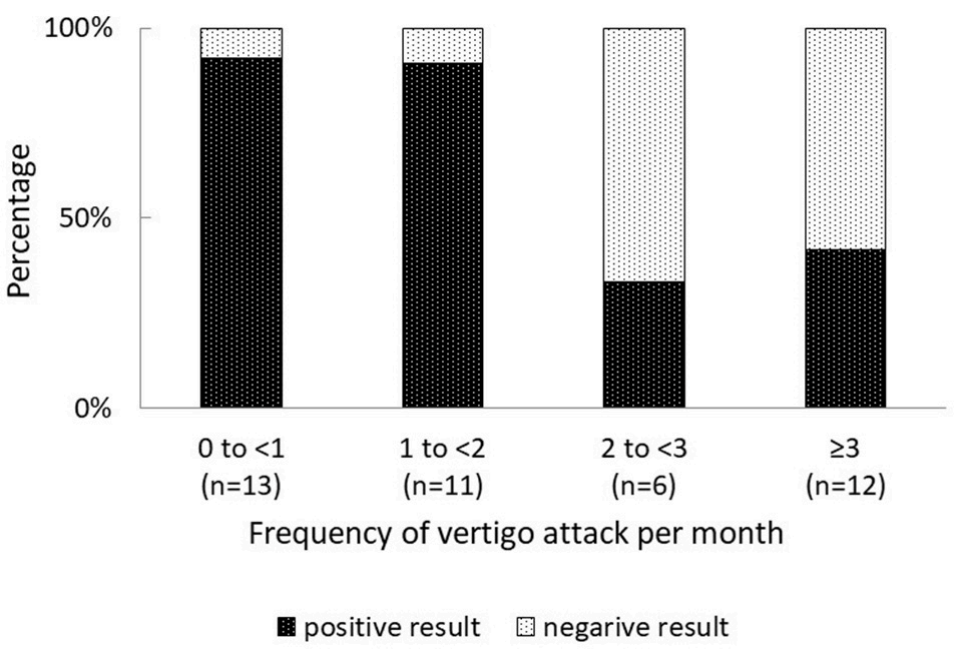
While the frequency of negative FVEMP test results was < 10% in cases of < 2 vertigo attacks per month, it was more than 50% in cases of ≥ 2 vertigo attacks per month. The results of the examination were more likely to be negative in cases of frequent vertigo attacks.

Overall, 31% (13 of 42) patients with MD had negative FVEMP results (Table 1). In the univariate analysis, age, sex, affected side, stage, disease duration, and mean PTA in lower, middle, and higher frequencies did not significantly associate to the results on FVEMP testing (Table 2). In contrast, the number of days since the last vertigo attack significantly associated to the results [odds ratio (OR): 1.07, 95% confidence interval (CI): 1.01–1.13, *p* = 0.031]. A shorter interval since the last vertigo attack associated to a negative FVEMP result. Figure 1 shows the results in each group per 7 days since the last vertigo attack. When < 7 days had passed since the last attack, the ratio of negative results was over 0.5. Furthermore, the frequency of vertigo attacks per month associated to the results on FVEMP (OR: 0.42, 95%CI: 0.23–0.75, *p* = 0.003). Figure 2 shows the results in each group per number of attacks. The more frequent the attacks, the more likely that the FVEMP result would be negative; a negative rate was over 0.5 in cases of two or more attacks per month.

**Table 1:** Background of subjects.

		**Results of FVEMP testing**		
		**Negative (*n* = 13)**	**Positive (*n* = 29)**	***p*-value**
Age		48.00 ± 14.01	52.34 ± 11.63	0.300
Sex	Female	7 (53.8%)	12 (41.4%)	0.516
	Male	6 (46.2%)	17 (58.6%)	
Affected side	Left	9 (69.2%)	13 (44.8%)	0.19
	Right	4 (30.8%)	16 (55.2%)	
Stage	1	4 (30.8%)	9 (31.0%)	1
	2	2 (15.4%)	5 (17.2%)	
	3	7 (53.8%)	14 (48.3%)	
	4	0 (0.0%)	1 (3.4%)	
Period since the last vertigo attack (days)		11.08 ± 15.50	25.79 ± 18.37	0.016
Frequency of vertigo attacks per month		2.88 ± 1.50	1.25 ± 1.15	< 0.001
Disease duration (months)		32.54 ± 50.72	34.86 ± 53.64	0.896
**Mean PTA threshold (dB)**				
	On 125 and 500 Hz	41.92 ± 21.31	43.62 ± 17.11	0.784
	On 500, 1,000 and 2,000 Hz	39.74 ± 22.18	39.77 ± 20.35	0.997
	On 4,000 and 8,000 Hz	42.31 ± 26.58	44.48 ± 24.45	0.797

*FVEMP, furosemide-loading vestibular evoked myogenic potential; PTA, pure tone audiometry*.

**Table 2:** Results of univariate analyses.

**Factor**	**OR (95%CI)**	***p*-value**
Age	1.03 (0.98–1.09)	0.294
Male sex	1.65 (0.44–6.17)	0.455
Left ear	2.77 (0.69–11.1)	0.150
Stage 1	(Reference)	
Stage 2	1.11 (0.15–8.37)	0.919
Stage 3 and 4	0.95 (0.22–4.19)	0.950
Period since the last vertigo attack (days)	1.07 (1.01–1.13)	0.031
Frequency of vertigo attacks per month	0.42 (0.23–0.75)	0.003
Disease duration (months)	1.00 (0.96–1.01)	0.893
**Mean PTA threshold (dB)**		
On 125 and 500 Hz	1.00 (0.96–1.03)	0.778
On 500, 1,000 and 2,000 Hz	1.00 (0.97–1.03)	0.997
On 4,000 and 8,000 Hz	1.00 (0.97–1.02)	0.791

*PTA, pure tone audiometry; OR, odds ratio; CI, confidence interval*.

Based on these results, right ear, days since the last vertigo attack < 7 and frequency of vertigo attacks per month ≥ 2 were entered in the multivariate analysis. It was found that both days since the last vertigo attack < 7 (OR: 0.13, 95% CI: 0.02–0.88, *p* = 0.039) and frequency of vertigo attacks per month ≥ 2 (OR: 0.06, 95% CI: 0.009–0.42, *p* = 0.004) were risk factors for a negative result on FVEMP testing (Table 3).

**Table 3:** Results of multivariate studies.

**Factor**	**OR (95%CI)**	***p*-value**
Right ear	3.22 (0.51–20.5)	0.214
Days since the last vertigo attack <7	0.13 (0.02–0.88)	0.039
Frequency of vertigo attacks per month ≥2	0.06 (0.009–0.42)	0.004

*OR, odds ratio; CI, confidence interval*.

## Discussion

Interestingly, results of FVEMP testing were negative in patients with recent and frequent vertigo attacks, although positive results suggest the presence of EHs (6–8). In order to elucidate this intuition countermeasure, several issues must be discussed.

First, we consider the effects of furosemide on the vestibular organs. The endolymphatic potential (EP) of the stria vasularis in the cochlea is reduced after furosemide administration, which transiently affects ototoxity. However, the EP of the semicircular canal ampullar wall minimally changed after furosemide administration (18). The amplitude of the short-latency vestibular-evoked response, which initiated in the otolith organ, and those that initiated in the semicircular canal, changed minimally after furosemide administration in the cat surface electrode study (19). Therefore, furosemide has little effect on vestibular function, unlike its impact on cochlear function. In addition, furosemide somewhat relieves vestibular hydrops in experimental animal models, although not as much as cochlear hydrops (20). While there is a little evidence that furosemide reduces EH, the vestibular function recovers to normal after furosemide administration in patients with MD (5, 6, 8, 11). We suspect that the phenomenon is due to reduced EH by the dehydration effect of furosemide in the vestibular system.

Second is the significance of acquiring positive results on FVEMP testing. The examination involved stimulation using tone burst sounds at 500 Hz, where the maximum response frequency on cVEMP in normal subject is (11). Due to EH, the resonance of the saccule shifts to higher frequencies and the response at 500 Hz apparently decreases in cases of MD (21, 22). After furosemide administration, the altered tuning recovers to near normal and the response at 500 Hz recovers (11, 22). A positive finding on FVEMP testing in patients with MD depends on the recovery of the altered resonance of the saccule.

Third, we consider the essential state of vertigo attacks in MD. Undoubtedly, EH presence is an important finding in MD; however, not all individuals with EH exhibit clinical symptoms of the disease (13, 23). Lindsay et al. described a simple pressure on the sensory epithelium due to the EH cause the vestibular dysfunction (24). However, animal models with experimentally created EHs showed hearing disturbance but no pathological nystagmus (25). Additional mechanisms are required to induce vertigo attacks in patients with EH. Schuknecht speculated that rupture of the membranous labyrinth is the direct cause of vertigo attacks in MD (26). Rupture of the membranous labyrinth is a specific finding in temporal bones with EHs. It is more often seen in patients with EH and symptoms of MD than in those with EH and without symptoms of MD (23, 27). Due to the discontinuity of the membranous labyrinth, the contamination of the perilymphatic space with potassium-rich endolymph causes impairment of the vestibular sensory cells. Experimental infusion of artificial endolymphatic fluid to the perilymphatic space led to transient nystagmus resembling an MD vertigo attack in an animal study (28). Thus, rupture of the membranous labyrinth theory is classic but has still been accepted as a cause of vertigo attacks in MD (29).

Based on the above evidence, the apparently irrational result that was obtained in this study can be explained by the consequences of membranous labyrinth rupture during vertigo attacks. Owing to the communication between the endolymphatic and perilymphatic spaces, furosemide does not reduce endolymphatic hydrops; thus, the altered resonance of the saccule remains, even after the administration, and the FVEMP test results are negative. Although the ruptures are more frequent in the membranous labyrinth of the cochlear than in the saccule, both organs communicate with each other via the canalis reunion (27). Thus cochlea rupture prohibits the reduction of saccular hydrops upon furosemide administration. Also, upon contamination of the endolymph and perilymph, the saccular sensory cells are impaired (26). Since this does not improve upon furosemide administration, the FVEMP results are negative. Several days after the vertigo attack, when the ruptured membrane has been repaired, the endolymph reaccumulates, and furosemide reduces endolymphatic hydrops, the FVEMP test results will be positive (26). This line of reasoning is sufficient to justify the irrational results of our study.

Young et al. reported that while abnormal cVEMP results were seen in 17% of patients with stage 1 disease and 60% with stage 4 disease; thus, the cVEMP results depend on the stage of MD (30). With the progression of MD, the inner ear sensory hair cells degenerate, resulting in cVEMP abnormalities. On the other hand, the FVEMP result was not related to stage in our study. This indicates that EH is generally present, even as the stage progresses, in MD.

Another point to note is the tuning property of cVEMP. As mentioned above, the frequency response in MD is displaced to high frequency, compared to that of normal subjects (21, 22). Based on this phenomenon, Murofushi et al. proposed the tuning property test to screen for MD (31). When the tuning property index was calculated from the amplitude of the p13-n23 wave of cVEMP at 500 Hz and at 1000 Hz as −19.9, the sensitivity was 74% and specificity was 0.76 for MD screening. Compared to FVEMP, this method does not require invasive drug administration. The result depends on the frequency displacement of cVEMP. While the phenomenon is highly specific to MD, the effects of other pathogenesis are unknown. In this respect, FVEMP is better than the tuning property method because it predicts EH more directly due to diuretic load.

It is well known that MD has a female pre-dominance (32). However, the number of females was 19, and that of males was 23, in this study. Was there any bias in subject selection? According to Minor et al., the female:male ratio was 1.3:1; thus, the ratio of females was 0.565 (32). Also, the 95% confidence interval of the female ratio was 0.298–0.613 in this study. Statistically, the ratio that was observed in this study is not different from previously reported ratios of MD populations. The dominance of males in this study does not indicate that there was bias in the selection of subjects.

This study was performed to detect the factors which influent the results of FVEMP. We resulted that as patients in various states are included in clinical study samples, it may be unproductive to discuss the positive rate in MD. More importantly, the interesting finding derived from this study is that FVEMP testing can detect EH and the condition of the membranous labyrinth. Can the result predict the clinical course of the future? Indeed we had previously reported that positive result of FVEMP related to the development of bilateral MD. Further study is required to clarify how the results of FVEMP are useful for future prediction. Note that FVEMP testing aimed at detecting EH may produce unreliable results when it is performed within 7 days of a vertigo attack or in patients who experience two or more vertigo attacks per month.

## Ethics statement

All experiments followed the tenets of the Declaration of Helsinki. This study was approved by the Institutional Review Board of Kindai University Faculty of Medicine.

## Author contributions

TS designed the study, interpreted the data, and wrote the manuscript. TS, TF, KSa, and KD collected the data. TK and KSh performed the statistical analysis. All authors approved the final version and agree to be accountable for this work.

### Conflict of interest statement

The authors declare that the research was conducted in the absence of any commercial or financial relationships that could be construed as a potential conflict of interest.
